# Syndrome de la personne raide associé à une dermatite herpétiforme: à propos d’un cas

**DOI:** 10.11604/pamj.2021.40.27.30313

**Published:** 2021-09-09

**Authors:** Loubna Tayebi, Ansumana Mohammed Keita, Nisrine Louhab, Mouna Zahlane, Laila Benjilali, Lamiaa Essaadouni

**Affiliations:** 1 Service de Médecine Interne, Hôpital Arrazi Centre Hospitalier Universitaire Mohamed VI, Marrakech, Maroc,; 2 Service de Neurologie, Hôpital Arrazi Centre Hospitalier Universitaire Mohamed VI, Marrakech, Maroc

**Keywords:** Syndrome de la personne raide, anti-GAD65, dermatite herpétiforme, à propos d’un cas, Stiff person syndrome, anti-GAD65, dermatitis herpetiformis, case report

## Abstract

Le syndrome de la personne raide (SPR) est une maladie rare affectant le système nerveux central et qui peut être d´origine auto-immune, paranéoplasique ou idiopathique. Sa présentation classique typique est caractérisée par une rigidité progressive du tronc et des membres, associée à des spasmes. Le diagnostic est soutenu par l'existence d'une activité musculaire continue et spontanée en détection à l'électroneuromyogramme, la présence d'anticorps anti-acide glutamique décarboxylase (anti-GAD) sériques, et une réponse aux benzodiazépines. Nous rapportons le cas d'un patient de 46 ans ayant une forme classique de syndrome de la personne raide auto-immune associée à une dermatite herpétiforme.

## Abstract (en)

Stiff person syndrome (SPS) is a rare disease affecting the central nervous system which can be autoimmune, paraneoplastic or idiopathic in origin. Its typical classic presentation is characterized by progressive stiffness of the trunk and limbs, associated with spasms. The diagnosis is supported by the existence of continuous and spontaneous muscle activity on electroneuromyogram detection, the presence of serum anti-GAD antibodies, and a response to benzodiazepines. We report the case of a 46-year-old patient with a classic form of autoimmune stiff person syndrome associated with dermatitis herpetiformis.

## Introduction

Le syndrome de la personne raide (SPR) est une maladie rare affectant le système nerveux central et se manifeste par une raideur, des spasmes musculaires et une sensibilité accrue aux stimuli externes qui aggravent encore les contractions [1]. Il a été décrit pour la première fois en 1956 par Moersch et Woltman qui ont analysé 14 patients avec une rigidité axiale associée à des spasmes douloureux [2]. Il s´agit d´une maladie de diagnostic difficile et souvent tardif, avec une évolution progressive et grave aboutissant au décès qui survient habituellement 6 à 15 ans après le début [3]. Nous rapportons l´observation d´un patient atteint de syndrome de la personne raide concomitant à une dermatite herpétiforme.

## Patient et observation

**Informations du patient:** un patient âgé de 46 ans, sans antécédents pathologiques notables, avait consulté dans notre formation pour bilan de rigidité axiale majeure, d´installation rapidement progressive. Il se plaignait il y´a 8 mois d´une raideur articulaire et de contracture musculaire douloureuse avec rigidité, associée à des lésions vésiculeuses cutanées prurigineuses. Ce tableau clinique s´est aggravé il y´a 3 mois par une dyspnée stade II de *New York Heart Association* (NYHA) avec dysphagie au solide et dysphonie sans altération d´état général ni fièvre.

**Figure 1:**
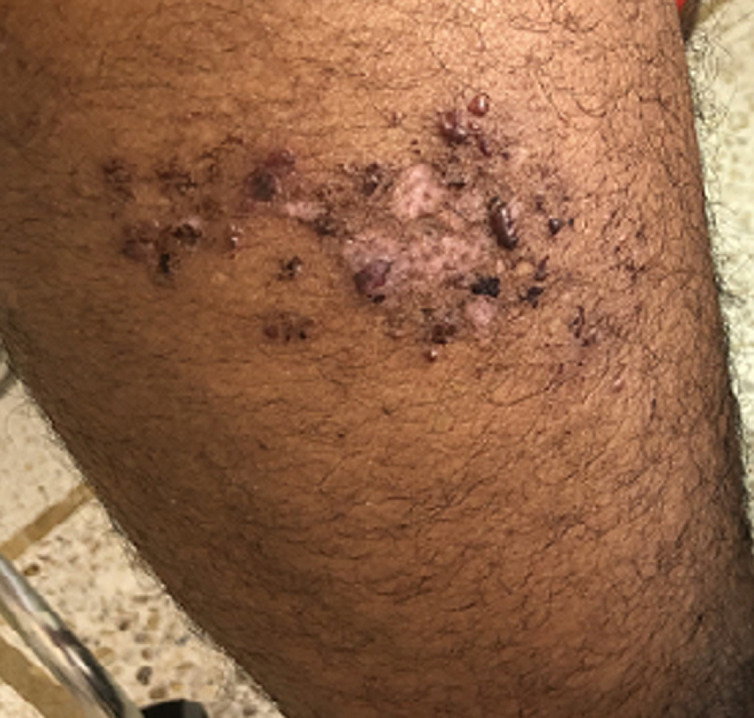
lésions cutanées vésiculo-bulleuses à contenu hémorragique de taille millimétrique à 1 cm de regroupement herpétiforme, localisées au niveau des faces postéro-latérales de la cuisse droite

**Figure 2:**
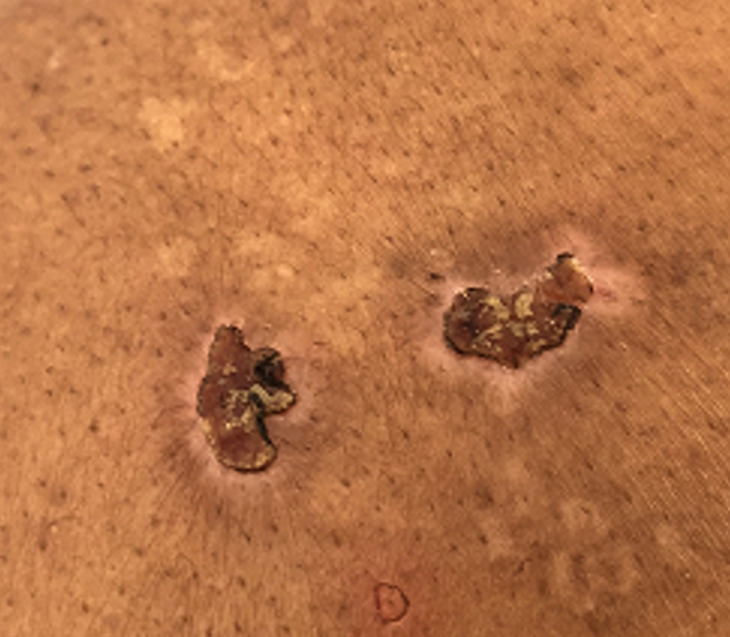
lésions crouteuses au niveau du dos

**Figure 3:**
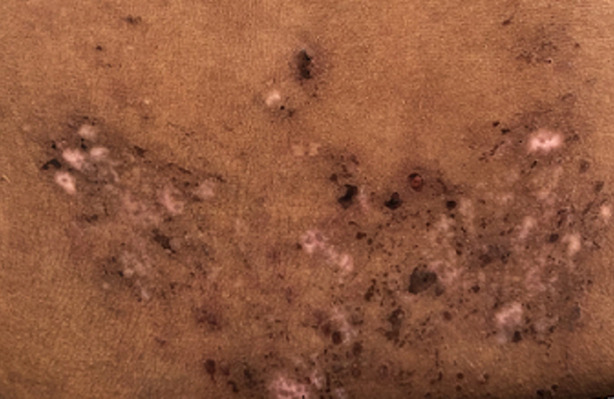
lésions cicatricielles au niveau de la région lombaire

**Figure 4:**
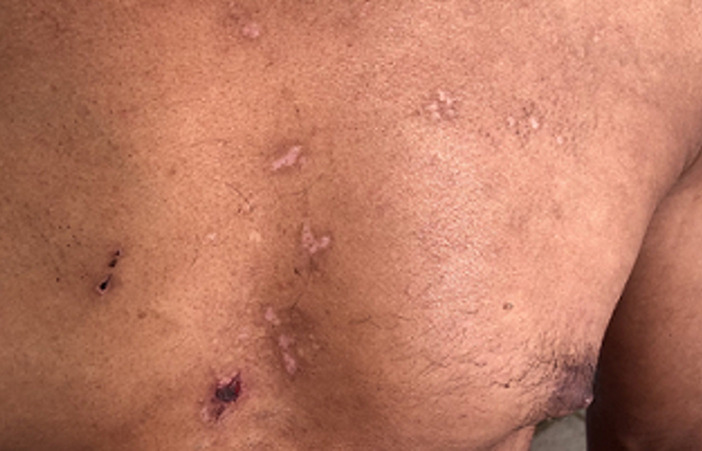
cicatrice de grattage au niveau de la partie supérieure du thorax

**Résultats cliniques:** l´examen clinique a objectivé des muscles hypertrophiés et rigides aux quatre membres avec signe de gowers positif, diminution de la protraction de la langue, les réflexes ostéo-tendineuses abolis aux membres inférieurs. L´examen ostéo-articulaire a montré une douleur à la mobilisation active et passive des grosses et petites articulations avec raideur au niveau des deux poignets, des pouces, des épaules et des chevilles, douleur à la palpation des épineuses lombaires avec lordose lombaire et raideur du rachis cervical. L´examen cutané a trouvé des lésions cutanées vésiculo-bulleuses à contenu hémorragique de taille millimétrique à 1cm, de regroupement herpétiforme, localisées au niveau des faces postéro-latérales des deux cuisses avec des lésions cicatricielles et des croutes au niveau du dos et de la partie supérieure du thorax associés à des lésions de grattage (Figure 1, Figure 2, Figure 3, Figure 4). Le reste de l´examen somatique a été sans particularités.

**Figure 5:**
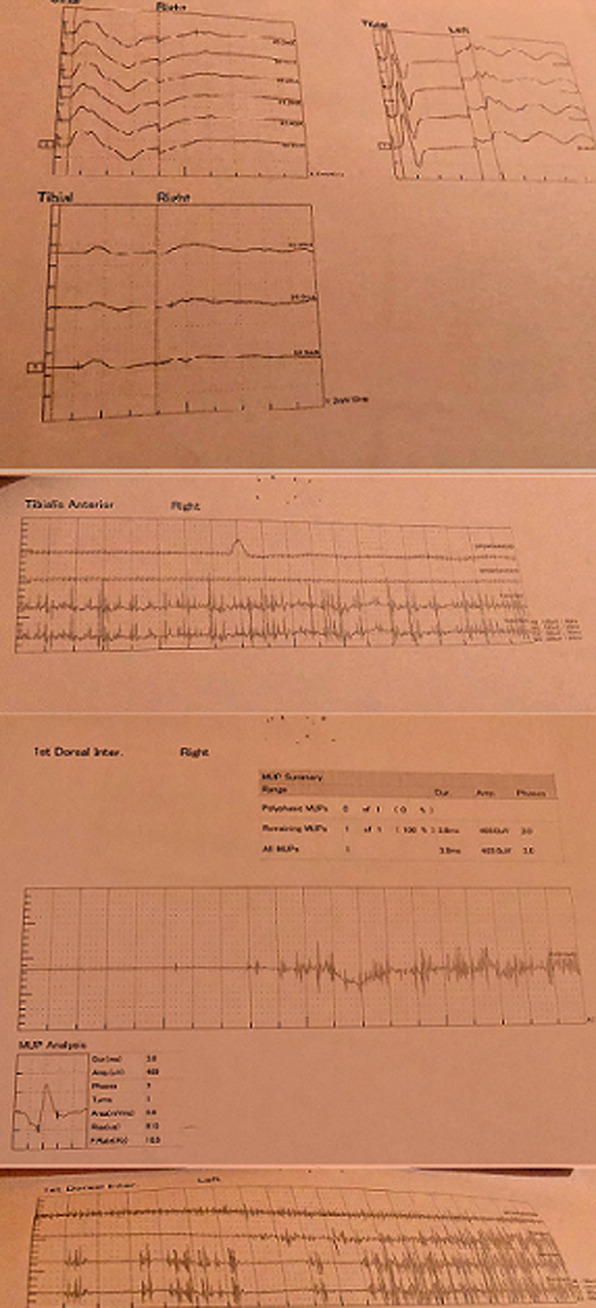
tracés EMNG; aspect en faveur d´un syndrome de la personne raide avec une activité musculaire au repos avec une décharge répétitive continue aux muscles paravertébraux, associée à un canal carpien bilatéral

**Démarche diagnostique:** les examens paracliniques ont objectivé des anticorps anti-acide glutamique décarboxylase (anti-GAD ) positif, les anticorps anti-amphiphysine, anticorps anti-nucléaires, anticorps anti-dna natif et anticorps anti-peptides cycliques citrullinés (anti-CCP) ont été négatifs. Les marqueurs tumoraux notamment le dosage de l´antigène prostatique spécifique (PSA) et l´alpha feotoprotéine ont été négatifs. Le scanner thoraco-abdo-pelvien a été normal. L´endoscopie digestive haute avec biopsie a montré un aspect de pangastrite chronique érythémateuse d´activité modérée, non atrophique, sans métaplasie intestinale ni dysplasie associée à la présence d´*Helicobacter pylori*, les plis duodénaux ont été légèrement effacés, avec une duodénite subaiguë et chronique non spécifique. Ainsi qu´une eosophagite subaiguë et chronique non spécifique, avec absence de signes de malignité. La naso-fibroscopie n´a pas révélé d´anomalie. La biopsie cutanée a objectivé une dermatite d´interface avec présence d´une fibrose dermique et excoriation, dépôts de C1q et faible Immunoglobulin G (IgG) en gains épais le long de la membrane basale. L´électromyoneurographie a objectivé une activité musculaire au repos avec une décharge répétitive continue aux muscles paravertébraux, associée à un canal carpien bilatéral. Cet aspect est en faveur d´un syndrome de la personne raide (Figure 5).

**Intervention thérapeutique et suivi:** vu la positivé de l´antiGAD65 chez notre patient, on a opté pour une benzodiazepine (valium) et le Baclofene associés à une rééducation motrice. La réponse thérapeutique était médiocre sur le plan clinique, ensuite on a mis le patient sous rituximab.

**Perspective du patient:** pendant l'hospitalisation et à la sortie, le patient était ravi des soins.

**Consentement du patient:** le consentement éclairé a été obtenu du patient pour que nous utilisions le cas.

## Discussion

Le syndrome de la personne raide est une pathologie rare qui touche l´adulte d´âge moyen (de la troisième à la septième décennie). Il est plus fréquent chez les femmes que les hommes [4]. Sa présentation clinique est classée en différents sous-types [5]: 1) syndrome de la personne raide de type classique; 2) syndrome de la personne raide focal ou segmenté; 3) syndrome de la personne raide avec spasmes; 4) variante d'encéphalomyélite évolutive avec raideur et myoclonie; 5) syndrome de la personne raide avec ataxie, épilepsie; 6) variante paranéoplasique. Notre patient a présenté un syndrome de la personne raide type classique retenu selon les critères diagnostiques résumés dans le Tableau 1 [5]. La pathogénie du syndrome de la personne raide s´explique par un blocage fonctionnel des synapses neuronales qui utilisent l'acide gamma aminobutyrique (GABA) 1 comme neurotransmetteur dans le cerveau et la moelle épinière, suite à la médiation du complexe immun anticorps et antigènes [6]. L'effet pathogène des anticorps anti-acide glutamique décarboxylase (anti-GAD65) est incertain, en raison de la localisation intracellulaire de l'antigène qui entrave l'action des anticorps et s'exprime avec différents syndromes cliniques [7].

**Tableau 1:** critères diagnostiques d´une forme typique du syndrome de la personne raide d'après Marinos C Dalakas 2009

Critères majeurs	Critères mineurs
Faciès inexpressifs avec raideur des muscles axiaux et des extrémités, principalement dans les muscles paravertébraux abdominaux et thoraco-lombaires qui provoquent une déformation constante de type hyperlordose, avec des difficultés respiratoires dues à l'implication des muscles thoraciques.	Anticorps sériques anti-GAD65 ou anti-amphiphysine positifs
Spasmes musculaires intermittents et douloureux causés par des stimuli environnementaux tels que le bruit, les stress émotionnels et tactiles.	Amélioration clinique avec les benzodiazépines
Tout compromis dû à d'autres maladies neurologiques doit être exclu.	
L'électromyogramme montre une activité motrice continue des muscles agonistes et antagonistes.	

L´anticorps anti-GAD65 est l´anticorps le plus fréquemment rapporté chez les patients atteints du syndrome de la personne raide. Cependant, les anticorps anti-GAD ne sont pas spécifiques du SPR, car jusqu'à 1% de la population normale peuvent les exprimer. Ils sont aussi présents chez 5% des différents syndromes neurologiques tels que l´ataxie cérébelleuse, l´encéphalite limbique avec myoclonie et l´épilepsie du lobe temporal localisée [7]. Notre patient avait des anticorps anti-GAD65 positifs. La gravité clinique de la maladie n'est pas liée au niveau des titres d'anticorps dans le sérum et dans le liquide céphalo-rachidien (LCR). Il y a des patients qui développent une maladie grave avec des titres bas et vice versa, tandis que d'autres qui ont la maladie ont des titres sériques négatifs [7]. L´atteinte cutanée fréquemment décrite en association avec le syndrome de la personne raide est le vitiligo [8]. Chez notre patient l´atteinte cutanée a été en faveur d´une dermatite herpétiforme qui est fréquemment associée à la maladie cœliaque [9], cependant notre patient a des anticorps anti transglutaminases IgA et IgG négatifs. On observe également une incidence élevée d´autres maladies auto-immunes associés à la dermatite herpétiforme, comme une thyroïdite, un lupus érythémateux systémique, une sarcoïdose, une anémie pernicieuse et un diabète ou parfois un lymphome intestinal [9].

Le traitement de syndrome de personne raide est abordé conformément à la positivité des anticorps identifiés comme positifs. Il existe des preuves de patients avec des anticorps positifs contre le récepteur GABA qui répondent mieux aux immunoglobulines. Les patients positifs à l'amphiphysine répondent mieux aux stéroïdes, à la plasmaphérèse et au traitement primaire du cancer. Ceux qui ont les anti-GAD positifs répondent mieux aux immunoglobulines, au diazépam, au clonazépam, tandis que les patients positifs à l´anti-GlyR alfa 1 ont des meilleurs résultats avec les immunothérapies. Le rituximab (anti-CD20) a donné dans les essais cliniques des réponses thérapeutiques avec une amélioration des spasmes et de la raideur musculaire, une bonne tolérance, de longues rémissions cliniques. Les doses allant de 350 à 375mg/m^2^administrés tous les 7 à 14 jours, ou chaque semaine, pendant 4 semaines.

## Conclusion

Le syndrome de la personne raide est une affection neurologique rare qui peut être d´origine auto-immune, paranéoplasique ou idiopathique. L´atteinte cutanée type dermatite herpétiforme n´est pas décrite en association au syndrome de la personne raide. Les traitements dépendent du profil immunologique et du terrain.
